# Boosting Photoelectrochemical Catalytic Ability of Bismuth Vanadate toward Water Oxidation via Synergistic Surface Defect Engineering and MXene-Assisted Charge Transport

**DOI:** 10.1021/acsomega.5c06250

**Published:** 2025-08-27

**Authors:** Tsai-Mu Cheng, Yi-Ru Wang, Yu-Hsuan Chiu, Chutima Kongvarhodom, Muhammad Saukani, Sibidou Yougbaré, Hung-Ming Chen, Lu-Yin Lin

**Affiliations:** † Graduate Institute for Translational Medicine, College of Medical Science and Technology, Taipei Medical University, Taipei 11031, Taiwan; ‡ Taipei Heart Institute, Taipei Medical University, Taipei 11031, Taiwan; § Cardiovascular Research Center, Taipei Medical University Hospital, Taipei 11031, Taiwan; ∥ Department of Chemical Engineering and Biotechnology, 34877 National Taipei University of Technology, Taipei 10608, Taiwan; ⊥ Department of Chemical Engineering, 65128 King Mongkut’s University of Technology Thonburi, 126 Pracha-u-thit, Toong-kru, Bangkok 10140, Thailand; # Department of Mechanical Engineering, Faculty of Engineering, Universitas Islam Kalimantan MAB, Jl. Adhyaksa No. 2, Banjarmasin 70124, Indonesia; ∇ 307955 Institut de Recherche en Sciences de la Santé (IRSS-DRCO)/Nanoro, 03 B.P 7192, Ouagadougou 03, Burkina Faso; ○ Gingen technology Co., LTD., Rm. 7, 10F., No.189, Sec. 2, Keelung Rd., Xinyi Dist., Taipei 11054, Taiwan

## Abstract

Bismuth vanadate (BiVO_4_) is regarded as a promising photoanode material for solar-driven photoelectrochemical (PEC) water oxidation, due to its visible-light absorption and favorable band edge positions. However, the practical application is hindered by limited charge carrier mobility and significant surface recombination. In this study, a dual-modification strategy is applied by combining alkaline etching and MXene integration to enhance surface reactivity and charge transport properties of BiVO_4_. Alkaline etching introduces structural defects and active sites on BiVO_4_ surface, which promote hole accumulation and facilitate interfacial redox reactions. Meanwhile, incorporating MXene forms a conductive interface that accelerates hole extraction and suppresses recombination. Although alkaline etching slightly reduces light absorption due to morphological restructuring, the subsequent MXene addition recovers and enhances photon harvesting. In the absence of hole scavengers, the pristine BiVO_4_ electrode achieves a photocurrent density of 4.65 mA/cm^2^ at 1.23 V vs RHE at AM 1.5G, which increases to 5.13 mA/cm^2^ for alkaline-etched BiVO_4_ and further to 6.15 mA/cm^2^ for alkaline-etched BiVO_4_ coupled with MXene (MXene/E-BVO). Moreover, the MXene/E-BVO electrode retains 93.4% of its initial photocurrent after continuous illumination for 10,000 s. These results confirm the effectiveness of combining surface and interfacial engineering to improve PEC water splitting performance of BiVO_4_.

## Graphical abstract

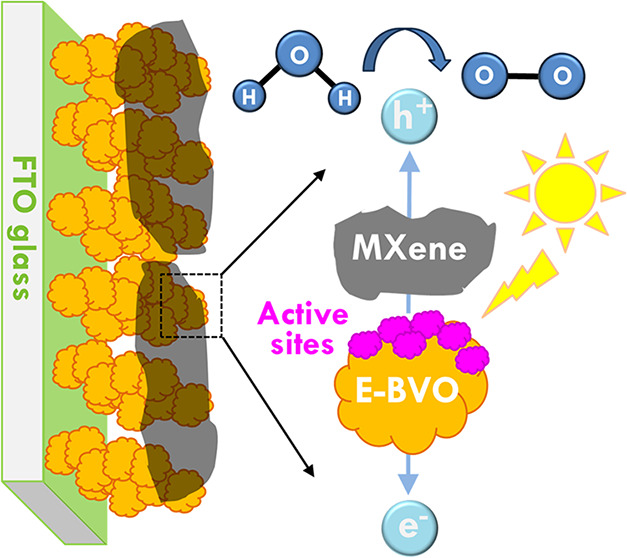

## 1 Introduction

Hydrogen is regarded as a clean energy carrier due to its high energy density and its generation of water as a byproduct.^1–3^ The transition from fossil fuel-based hydrogen production to renewable routes has gained significant momentum to global decarbonization goals and energy security demands.^4–6^ Among hydrogen generation strategies, photoelectrochemical (PEC) water splitting presents an attractive approach by enabling direct solar-to-hydrogen energy conversion through a combination of semiconductor photoabsorption, charge carrier generation, and redox catalysis at the semiconductor/electrolyte interface.^7–9^ This method has the potential to produce hydrogen sustainably without external electricity input to achieve sufficient efficiency and long-term operational stability.^10–14^

The photoanode in PEC water splitting plays a pivotal role by driving the oxygen evolution reaction (OER), which is kinetically sluggish and thermodynamically demanding.^15–19^ Effective photoanode materials must simultaneously achieve strong absorption of photons, efficient separation and transport of photogenerated charge carriers, and rapid interfacial electron transfer. Among the candidates, bismuth vanadate (BiVO_4_) has emerged as a promising visible-light-driven photoanode due to its suitable bandgap (∼2.4–2.5 eV), and appropriate valence and conduction band positions for water oxidation.^20–25^ Its monoclinic scheelite structure also favors carrier mobility in specific crystallographic directions. Nevertheless, the PEC performance of BiVO_4_ remains limited by poor charge transport within the bulk, rapid surface recombination of photogenerated holes, and insufficient catalytic activity toward OER. To overcome these limitations, several engineering strategies have been proposed, including elemental doping,^10,11,21,26^ cocatalyst deposition,^14,27^ nanostructuring,^28–30^ and heterojunction formation.^22,31,32^ Surface modification, in particular, has gained considerable attention because many performance-limiting processes in BiVO_4_ occur at the semiconductor/electrolyte interface.^33–35^ Among these, alkaline etching has proven effective in reconstructing the BiVO_4_ surface to expose additional active sites, increase surface area, and introduce oxygen vacancies or hydroxyl groups that facilitate hole trapping and water oxidation. Cheng et al. fabricated alkaline-etched BiVO_4_ by a hydrothermal method for catalyzing water oxidation. A photocurrent density of 2.38 mA/cm^2^ in Na_2_SO_4_ is obtained.^36^ Saada and co-workers reported electrodeposition of metal Bi in alkaline electrolyte for preparing photoelectrochemically active BiVO_4_ for catalyzing water splitting.^37^ Hsu et al. applied hydrothermal, soaking, and electrodeposition as different alkaline etching methods for conducting surface modifications on BiVO_4_ as the catalyst for water splitting with a photocurrent density of 2.38 mA/cm^2^ in a Na_2_SO_4_ solution.^38^ However, surface treatment alone often fails to address bulk conductivity and charge carrier transport, indicating the need for complementary strategies that target both surface and interfacial processes.

The integration of two-dimensional (2D) MXene materials such as Ti_3_C_2_T_*x*_ offers a promising route for improving interfacial charge transport. MXenes exhibit high electrical conductivity, rich surface functionality (e.g., −OH, –F, –O groups), and good processability in aqueous media, making them attractive for forming intimate contact with semiconductor surfaces.^25,39–42^ When used in photoelectrode systems, MXenes can act as hole extraction layers or conductive bridges to reduce series resistance, suppress electron/hole recombination, and accelerate interfacial charge transfer. Previous reports have demonstrated that MXene-functionalized BiVO_4_ composites exhibit improved PEC activity and photostability. Bai et al. constructed a hole transport layer on BiVO_4_ by doping MXene in Ferrihydrite, which presented a photocurrent density of 4.55 mA/cm^2^.^43^ Jahangir et al. synthesized Co_3_O_4_/MXene hybrids on BiVO_4_ as a dual-function material to catalyze water oxidation, and a photocurrent density of 5.05 mA/cm^2^ is obtained.^44^ Zhong and coauthors prepared metal–organic framework (MOF)/MXene/BiVO_4_ as a photocatalyst with a photocurrent density of 4.26 mA/cm^2^.^45^ However, most studies to date have applied MXene onto untreated or planar BiVO_4_ surfaces, potentially limiting the benefits of interfacial engineering due to insufficient surface area or poor coupling. The combination of alkaline etching and MXene integration has not been thoroughly explored as a synergistic approach to simultaneously enhance the surface activity and charge transfer capability of BiVO_4_ photoanodes. Alkaline etching introduces defect sites and increases roughness, providing favorable anchoring points for the conformal deposition of MXene layers. In turn, the ultrathin MXene layer can form intimate interfacial contact with the etched BiVO_4_ nanostructure, enabling rapid hole transport across the interface. This dual-modification strategy is expected to address both surface and interfacial bottlenecks in PEC water oxidation, leading to substantial improvements in photocurrent density and operational stability.

In this study, BiVO_4_ photoanodes were sequentially treated by alkaline etching and MXene incorporation to form a composite architecture. Morphological, structural, electronic and electrochemical properties of pristine BiVO_4_, alkaline-etched BiVO_4_ (E-BVO) and MXene coupled E-BVO (MXene/E-BVO) were analyzed to elucidate the effects of the dual treatments. The MXene/E-BVO photoanode exhibited a significantly enhanced photocurrent density of 6.15 mA/cm^2^ at 1.23 V vs RHE, which is much higher than the values obtained for pristine BiVO_4_ (4.65 mA/cm^2^) and E-BVO (5.13 mA/cm^2^) photoanodes. In addition, the MXene/E-BVO photoanode showed a low onset potential of 0.29 V vs RHE, a high carrier density of 4.12 × 10^24^ cm^–3^, and a reduced charge-transfer resistance of 116.6 Ω. Long-term durability tests confirmed 93.4% of the photocurrent retention after 10,000 s under continuous AM 1.5G illumination, highlighting the improved stability of the composite system. These results confirm that combining surface restructuring with conductive interfacial engineering offers a promising direction for advancing BiVO_4_-based photoanodes and PEC water-splitting technologies.

## 2 Experimental Section

### 2.1 Synthesis of Alkaline-Etched BiVO_4_ and That with MXene Incorporated Photoanodes

The pristine BiVO_4_ photoanode was prepared according to a previous work.^46^ Detailed experiments are shown in Supporting Information (SI). The alkaline-etched BiVO_4_ (E-BVO) photoanode is prepared by a hydrothermal method. A pristine BiVO_4_ photoanode is put into a liner which contains 0.1 M of NaOH solution (20 mL). The container is put into autoclave, which was then heated into 130 °C and maintained for 45 min. The product is washed by deionized water (DIW) after cooling down to obtain the E-BVO photoanode.

MXene powder (Ti_3_C_2_T_*x*_) was synthesized by selectively etching Al from Ti_3_C_2_T_*x*_ MAX phase using a modified HF-free LiF/HCl method. After etching, the MXene was washed repeatedly until the supernatant reached pH ∼ 6, and the resulting suspension was delaminated via ultrasonication in argon atmosphere. The concentration of the resulting MXene dispersion was adjusted to 1 mg/mL and stored under N_2_ to prevent oxidation. During MXene integration, E-BVO electrodes were immersed in the MXene solution in a Teflon-lined autoclave, allowing spontaneous coating driven by electrostatic attraction between negatively charged MXene nanosheets and the defect-rich BVO surface. The BVO and E-BVO photoanodes incorporated with MXene (MXene/BVO and MXene/E-BVO) are prepared by further conducting a hydrothermal method at 150 °C for 3 h with BVO and E-BVO photoanodes in the MXene solution. Scheme 1 shows a process to fabricate BVO, E-BVO, MXene/BVO and MXene/E-BVO.

**Scheme 1:**
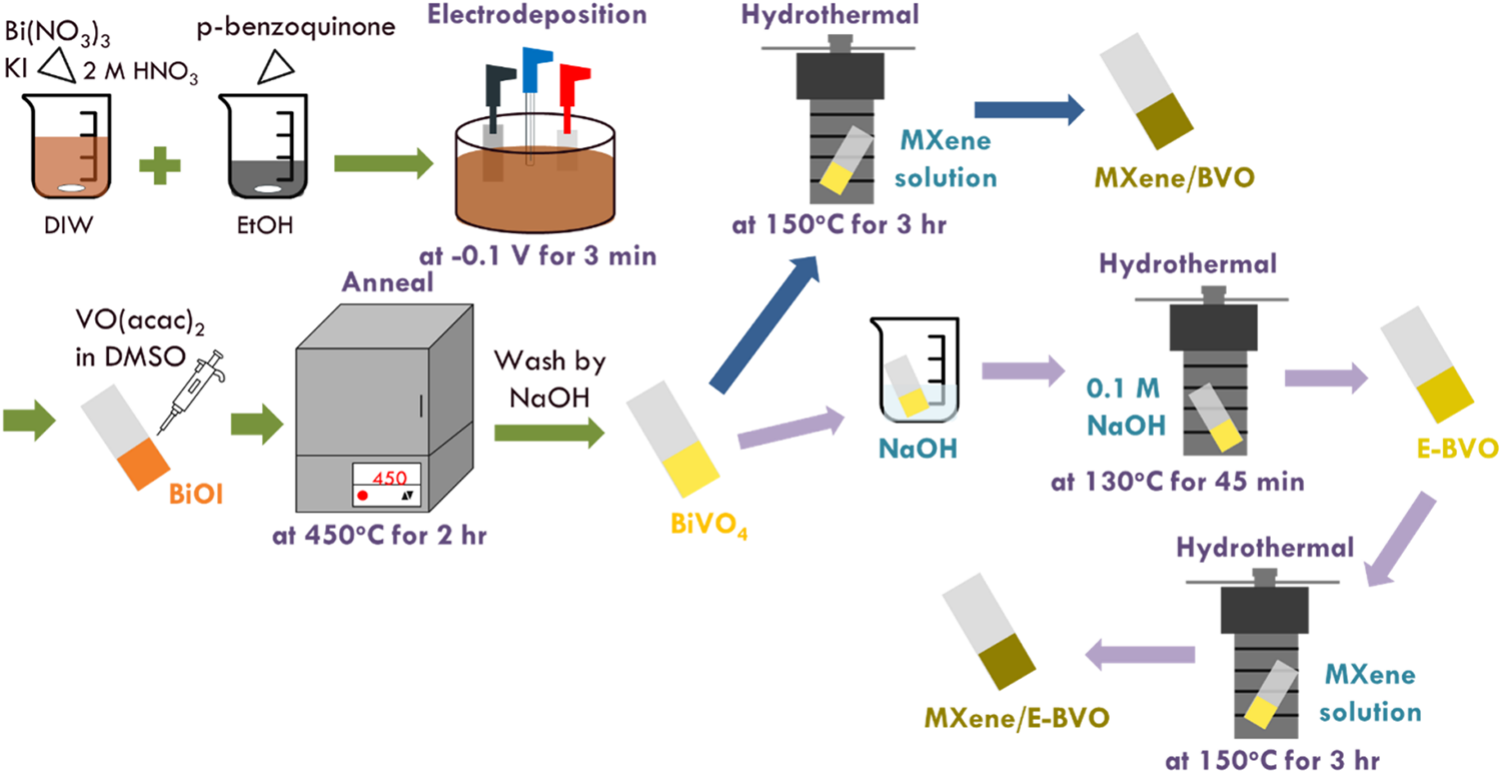
Process to Fabricate BVO, E-BVO, MXene/BVO and MXene/E-BVO

The temperature and duration of the hydrothermal process were optimized to balance the etching efficiency and preserve the rod-like BVO morphology. At 130 °C, sufficient surface reconstruction occurs without overdissolution, while at the subsequent 150 °C MXene treatment ensures intimate interfacial anchoring driven by thermal condensation and van der Waals interactions.

### 2.2 Fabrication of BVO Thin Film on FTO Substrate

The BVO photoanode was synthesized through a two-step method involving the electrodeposition of BiOI followed by a solution-based vanadium precursor conversion. Initially, BiOI was deposited on fluorine-doped tin oxide (FTO) glass using a three-electrode configuration, where Ag/AgCl and platinum wire served as the reference and counter electrodes, respectively. The deposition electrolyte was prepared by mixing two solutions. The first comprised 3.32 g of KI and Bi­(NO_3_)_3_·5H_2_O dissolved in deionized water, with the pH adjusted to 1.7 using nitric acid. The second solution consisted of 0.497 g of p-benzoquinone dissolved in 20 mL of absolute ethanol. Electrodeposition was performed at −0.1 V vs Ag/AgCl for 3 min. After deposition, a vanadium source solution was drop-cast onto the BiOI-coated substrate. This precursor solution contained 0.2 M vanadyl acetylacetonate and 0.02 M sodium tungstate dissolved in 5 mL of dimethyl sulfoxide (DMSO). The film was then annealed at 450 °C for 2 h using a ramp rate of 2 °C/min to form the final BVO structure. To remove surface residues such as V_2_O_5_, the annealed electrodes were rinsed with 1 M NaOH solution.

### 2.3 Characterization and Measurement Protocols

The surface morphology of the as-prepared photoanodes was examined by field-emission scanning electron microscopy (FE-SEM; Nova NanoSEM 230, FEI). Crystallographic features were analyzed using X-ray diffraction (XRD; X’Pert^3^ Powder, PANalytical), while elemental composition and chemical states were determined by X-ray photoelectron spectroscopy (XPS; VG ESCALAB 250, Al Kα source). Optical absorption properties and estimated band gaps were evaluated by UV–vis spectroscopy (JASCO V750). For electrochemical analysis, the photoelectrochemical measurements are carried out in a three-electrode cell with front-side illumination. The working electrode was mounted vertically facing the light source. A platinum wire served as the counter electrode, and an Ag/AgCl (saturated KCl) electrode was used as the reference. The electrolyte was 0.5 M Na_2_SO_4_ aqueous solution. Simulated sunlight was provided by a 300 W Xe arc lamp equipped with an AM 1.5G filter delivering an irradiance of 100 mW/cm^2^. The illuminated area of the photoanode is defined to 1 cm^2^. All electrodes were connected to an electrochemical workstation, and measurements were performed under ambient temperature without stirring. The photo for the setup was shown in Figure S1 in the SI. In our setup, the three electrodes are arranged in series along the light path, with the cathode closest to the illumination source, followed by the Ag/AgCl reference electrode, and the Pt counter electrode positioned last. This arrangement was chosen to maintain a compact and stable configuration within the photoelectrochemical cell. Although the reference electrode is illuminated, no abrupt potential fluctuations were observed during the measurements, as verified by stable baseline readings and consistent photocurrent responses. All measured potentials were converted to the reversible hydrogen electrode (RHE) scale by eq 1.^14^

1 E(vs.RHE)=E(vs.Ag/AgCl)+0.05916×7+0.197=E(vs.Ag/AgCl)+0.611

Electrochemical tests including linear sweep voltammetry (LSV) and electrochemical impedance spectroscopy (EIS) were conducted using a PGSTAT204 potentiostat/galvanostat (Metrohm Autolab) with an FRA2 module. All measurements were repeated at least three times to ensure reproducibility and minimize experimental deviation. Each synthetic condition was repeated at least three times to ensure reproducibility. Control samples without NaOH etching or without MXene addition were fabricated and characterized under the same protocols for comparison.

## 3 Results and Discussion

### 3.1 Morpholgoy and Composition Examinations of BVO, E-BVO, MXene/BVO and MXene/E-BVO

To examine the morphological evolution resulting from alkaline etching and MXene incorporation on BVO, the SEM images of BVO, E-BVO, MXene/BVO and MXene/E-BVO are presented in Figure 1(a,e), (b,f), (c,g), and (d,h), respectively. Figure S2 in the SI shows the TEM image of MXene/E-BVO. The pristine BVO exhibits a uniform rod-like array, with individual rods showing slight curvature and polycrystalline features along their axis. The average diameter of these rods is around 150 nm. Upon alkaline etching, E-BVO displays thicker and more interconnected rods, implying that localized dissolution and redeposition occur during the treatment. This process likely promotes the fusion of adjacent rods, resulting in a denser morphology with reduced spacing between structures. While narrowed inter-rod gaps may suppress light penetration into deeper regions, the enhanced connectivity could improve charge transport efficiency across the photoanode. After MXene incorporation, an ultrathin and uniform layer is seen covering the BVO and E-BVO surface. The original rod-like structure is well-preserved, indicating that MXene addition does not interfere with the BVO morphology. The conductive MXene coating is expected to facilitate hole extraction and suppress surface charge recombination, thereby contributing to improved interfacial charge transfer kinetics. This surface modification is thus anticipated to enhance the overall photoelectrochemical performance in water oxidation reactions.

**Figure 1:**
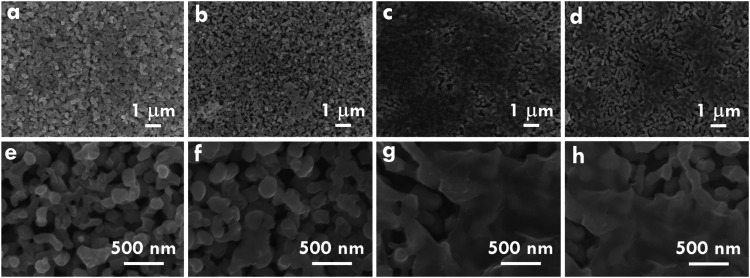
SEM figures of (a, e) BVO, (b, f) E-BVO, (c, g) MXene/BVO and (d, h) MXene/E-BVO.

The phase compositions of BVO, E-BVO, MXene/BVO and MXene/E-BVO are examined through X-ray diffraction (XRD), with the corresponding patterns shown in Figure 2. All samples display diffraction peaks in the 2θ range of 15° to 35°, which correspond well to the (110), (011), (121), and (040) planes of monoclinic BiVO_4_ (JCPDS #14-0688),^47^ confirming that the desired crystal structure is maintained after alkaline etching and MXene addition. Peaks originating from the SnO_2_ layer (JCPDS #41-1445)^48^ are also detected, which stem from the fluorine-doped tin oxide (FTO) substrate commonly used for growing BVO photoanodes. These substrate-derived peaks serve as internal references and do not interfere with the assignment of BiVO_4_ phases due to their well-separated positions. No new diffraction signals are observed in the E-BVO, MXene/BVO and MXene/E-BVO samples, indicating that the structural integrity of the BiVO_4_ lattice remains intact following postsynthetic treatments. Characteristic MXene reflections are not visible, which can be attributed to its relatively low loading and possible delamination. A subtle peak shift near 2θ value of 28.9° (assigned to the (121) plane) is evident for MXene/BVO and MXene/E-BVO (Figure 2b), implying that interfacial interactions between MXene and BVO may introduce lattice strain or local distortion that can enhance charge separation and catalytic performance.^49^ This phenomenon likely arises from interfacial interactions between MXene sheets and the BVO surface during the post-treatment process. The negatively charged MXene surface, rich in functional groups such as −OH, –O, and –F, may interact with the surface Bi or V atoms via hydrogen bonding or electrostatic interactions. These interactions can introduce localized stress or distortion in the adjacent BiVO_4_ lattice, leading to a change in interplanar spacing and thus a measurable shift in XRD peak position. Such strain effects have been reported in oxide-MXene hybrids and are often associated with enhanced surface reactivity, modified band structure, or improved charge separation. All factors are beneficial for photoelectrochemical performance.

**Figure 2:**
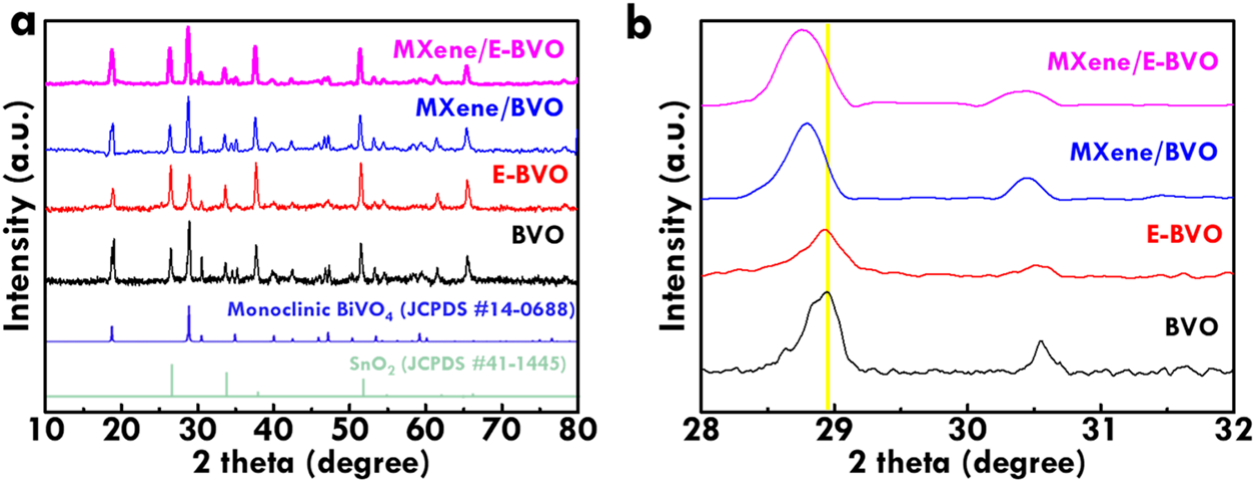
XRD patterns of BVO, E-BVO, MXene/BVO and MXene/E-BVO in (a) wide and (b) narrow degree.

To investigate the chemical compositions and oxidation states in greater detail, X-ray photoelectron spectroscopy (XPS) measurements were conducted, and the corresponding spectra are shown in Figure 3. The Bi 4f spectra for BVO, E-BVO, MXene/BVO and MXene/E-BVO are shown in Figure 3a–d, respectively. Each spectrum presents two split orbitals of Bi 4f_7/2_ and Bi 4f_5/2_, centered at 158.6 and 163.9 eV, respectively. Peaks corresponding to Bi^3+^ and Bi^5+^ oxidation states are observed in all samples, with negligible shifts in binding energy, indicating that the electronic environment of Bi remains relatively unchanged after alkaline etching and MXene incorporation. The V 2p spectra of BVO, E-BVO,, MXene/BVO and MXene/E-BVO are presented in Figure 3e–h. The V 2p_3/2_ and V 2p_1/2_ peaks are respectively located near 516.8 and 524.3 eV, and are fitted with mixed V^4+^ and V^5+^ states. Similar intensity ratios and binding energies are found for all samples, suggesting minimal impact on vanadium chemistry. The O 1s spectra (Figure 3i–l show two deconvoluted peaks attributed to surface hydroxyl groups (OH^–^) at 531.4 eV and lattice oxygen (M-O) at 529.6 eV. The M-O component corresponds to metal–oxygen bonding within the BiVO_4_ structure. Finally, the Ti 2p spectra of MXene/BVO and MXene/E-BVO respectively shown in Figure 3m–n exhibits Ti 2p_3/2_ and 2p_1/2_ peaks respectively at 458.6 and 464.3 eV, assigned to Ti^3+^ and Ti^4+^ oxidation states. This confirms the successful incorporation of MXene into the composite. Based on XPS quantification, the atomic ratio of Ti to V in MXene/BVO and MXene/E-BVO is approximately 0.05 and 0.06, respectively, further validating the presence of MXene despite its low content.

**Figure 3:**
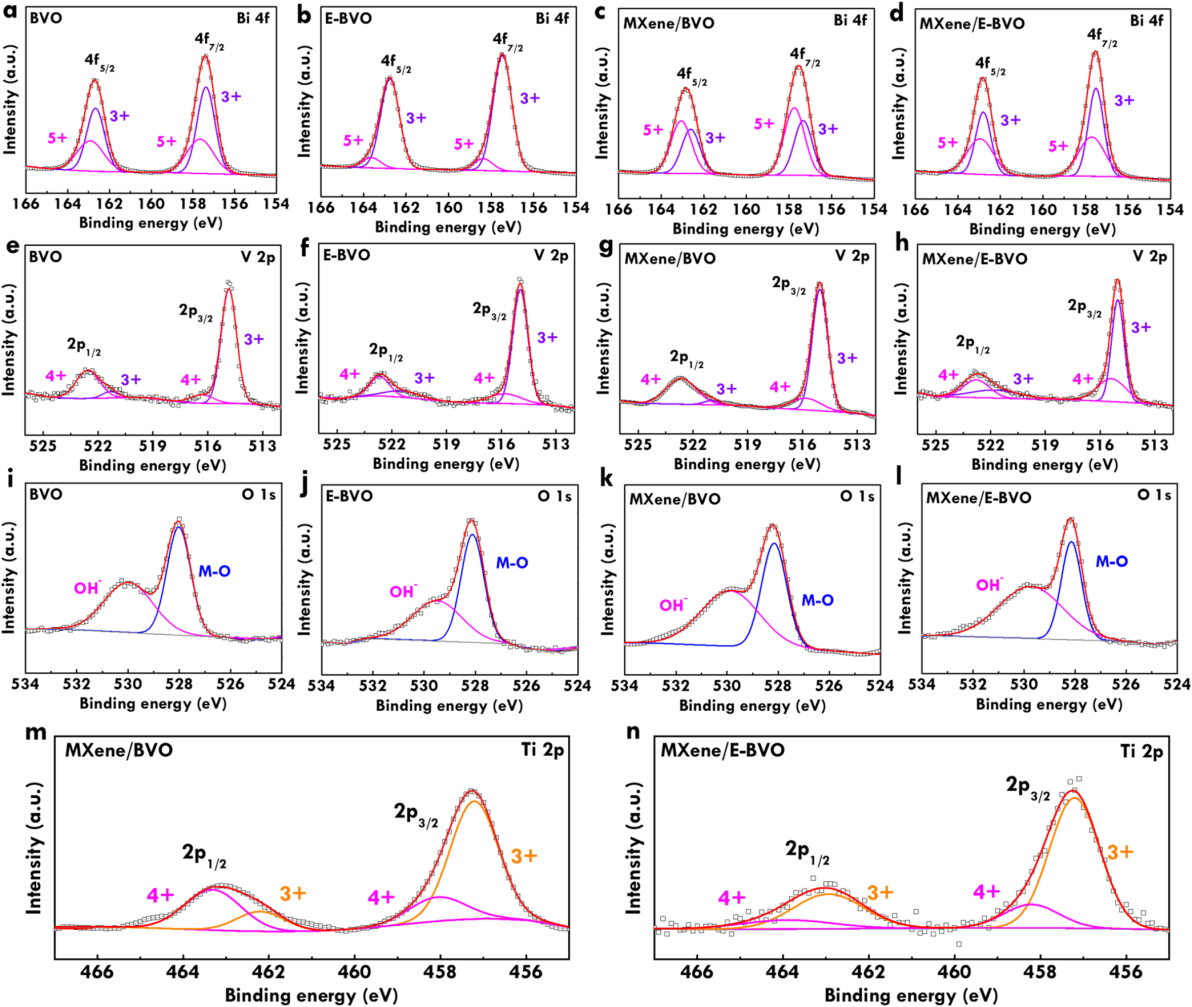
Bi 4f spectra of (a) BVO, (b) E-BVO, (c) MXene/BVO and (d) MXene/E-BVO; V 2p spectra of (e) BVO, (f) E-BVO, (g) MXene/BVO and (h) MXene/E-BVO; O 1s spectra of (i) BVO, (j) E-BVO, (k) MXene/BVO and (l) MXene/E-BVO; Ti 2p spectra of (m) MXene/BVO and (n) MXene/E-BVO.

The light absorption behaviors of BVO, E-BVO, MXene/BVO and MXene/E-BVO are analyzed by UV–vis spectroscopy, as shown in Figure 4a. All samples exhibit a pronounced absorption edge near 500 nm, characteristic of monoclinic BiVO_4_. Compared with the pristine BVO, the E-BVO sample shows a noticeable decrease in absorption intensity, which is likely attributed to morphological densification-specifically, the narrowed inter-rod spacing caused by alkaline etching that limits light penetration into deeper regions of the film. Interestingly, MXene/BVO and MXene/E-BVO samples recover much of the lost absorption, with intensity levels comparable to those of the pristine BVO. This improvement is possibly due to enhanced light scattering and surface plasmonic effects introduced by MXene sheets. To further assess the optical band structure, Tauc plots are derived from the absorbance spectra using the equation (α*h*ν)^2^ vs *h*ν (Figure 4b).^50^ The optical band gaps are extracted from the intercepts of the tangents, yielding values of 2.59, 2.55, 2.56, and 2.57 eV for BVO, E-BVO, MXene/BVO and MXene/E-BVO, respectively. These values are in close agreement with the reported band gap of ∼2.5 eV for monoclinic BiVO_4_.^51^ The minimal variations in band gap suggest that neither alkaline etching nor MXene incorporation significantly alters the bulk electronic structure. These results, combined with XPS findings, support the conclusion that the postmodification strategies primarily influence surface features and interfacial charge dynamics without disrupting the intrinsic band configuration of BVO.

**Figure 4:**
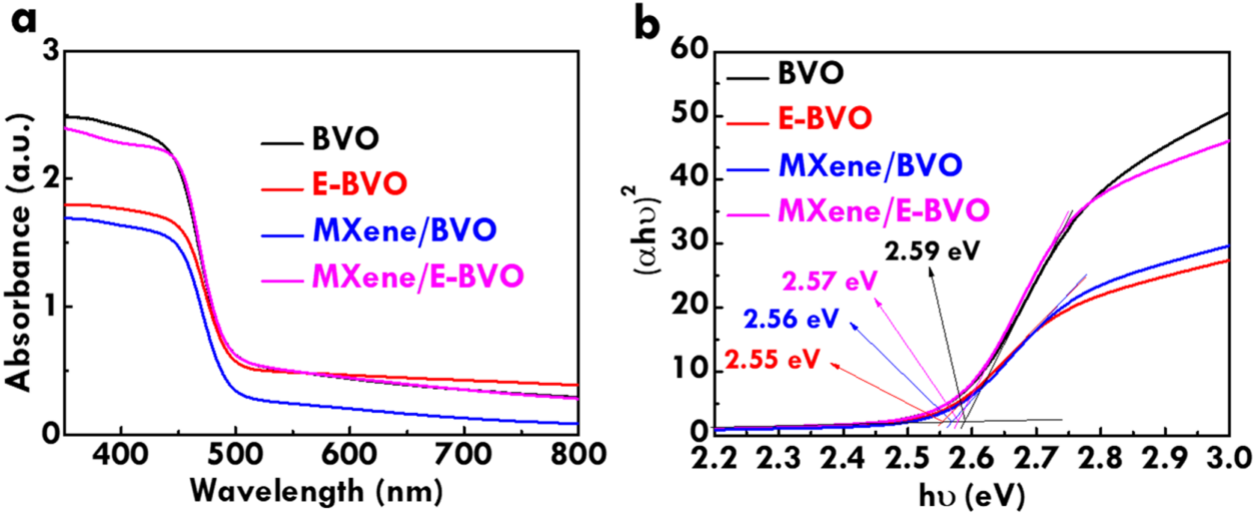
(a) The UV–vis spetrum and (b) Tauc plots of BVO, E-BVO, MXene/BVO and MXene/E-BVO.

### 3.2 Photoelectrochemical Catalytic Properties of BVO, E-BVO, MXene/BVO and MXene/E-BVO

The photoelectrochemical properties of BVO, E-BVO, MXene/BVO and MXene/E-BVO photoanodes were systematically analyzed to assess their light response, charge separation, and catalytic performance. Initially, chopped chronoamperometry measurements were performed at 1.23 V_RHE_ to evaluate photoresponsive behavior under intermittent light exposure (Figure 5a). Upon illumination, all three electrodes exhibit a sharp increase in photocurrent, confirming their photoactive nature. The current rapidly reaches a peak value; however, for both BVO and E-BVO, the current gradually decays within each illumination cycle, suggesting the presence of charge recombination. In contrast, the MXene/E-BVO electrode maintains a much more stable and higher photocurrent throughout the light-on periods, indicating improved charge separation and suppressed recombination. The dark currents quickly drop to zero for all samples, reaffirming the photoresponsive origin of the observed currents. Furthermore, across prolonged measurement durations, the photocurrent of MXene/E-BVO remains considerably stable, whereas BVO and E-BVO both show gradual decreases, suggesting better long-term operational stability imparted by MXene incorporation. This enhancement is attributed to the excellent electrical conductivity and interfacial contact provided by MXene, which facilitates more efficient hole extraction and charge transport.

**Figure 5:**
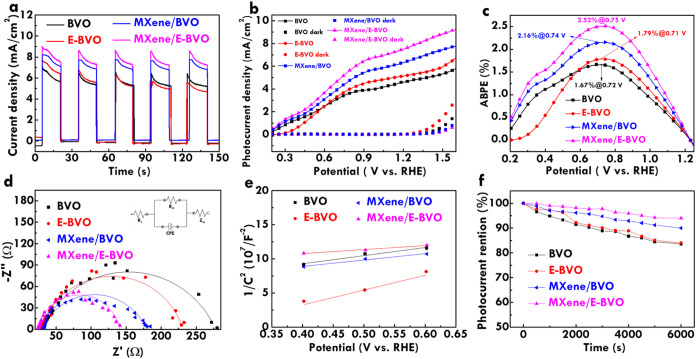
(a) Transient current figure at 1.23 V_RHE_; (b) LSV at 20 mV/s (illumination); (c) ABPE plots; (d) Nyquist figure at 1.23 V_RHE_ (illumination); (e) Mott–Schottky plots; (f) photocurrent retention and duration relationships of BVO, E-BVO, MXene/BVO and MXene/E-BVO electrodes.

The photoresponse characteristics were further evaluated using linear sweep voltammetry (LSV) under AM 1.5G illumination, with the results shown in Figure 5b. Table 1 shows the photocurrent density of BVO, E-BVO, MXene/BVO and MXene/E-BVO. The photocurrent densities at 1.23 V_RHE_ were determined to be 4.65, 5.13, 5.88, and 6.15 mA/cm^2^ for BVO, E-BVO, MXene/BVO and MXene/E-BVO, respectively. The increase in photocurrent for E-BVO compared to pristine BVO can be ascribed to the alkaline etching process, which induces surface restructuring and generates more catalytically active sites capable of enhancing hole accumulation and interfacial water oxidation. This enhancement occurs despite a reduction in optical absorption for E-BVO, indicating the greater influence of surface catalytic effects over light absorption in this case. The morphological restructuring of BVO induced by alkaline etching plays a significant role in enhancing its PEC performance. SEM analysis reveals that etching transforms the loosely packed rod-like arrays of pristine BVO into a denser and more interconnected network in E-BVO. This densification likely arises from partial dissolution and redeposition, which promotes fusion between adjacent rods and reduces inter-rod spacing. The tighter structure enhances electrical connectivity across the photoanode surface, facilitating more efficient in-plane charge transport and minimizing the risk of carrier trapping at grain boundaries. In addition, the increased surface roughness and interfacial area introduced by the etched morphology generate more catalytically active sites for water oxidation, promoting hole utilization and suppressing surface recombination. However, the morphological densification also slightly reduces light penetration due to narrowed voids, which can decrease overall light absorption, as confirmed by UV–vis measurements. Nevertheless, this optical loss is compensated by improved charge extraction and higher surface activity. The trade-off between light absorption and charge transport highlights the importance of morphological optimization in achieving a balance between photon harvesting and catalytic efficiency. Moreover, the MXene/E-BVO electrode exhibits the highest photocurrent density, confirming that the incorporation of MXene introduces additional conductive pathways and promotes rapid charge transfer across the interface. Onset potentials were also determined to be 0.25, 0.31, 0.22, and 0.20 V_RHE_ for BVO, E-BVO, MXene/BVO and MXene/E-BVO, respectively. The higher onset potential of E-BVO may originate from increased defect levels that elevate the overpotential required for water oxidation. Conversely, the significantly reduced onset potential in MXene/E-BVO suggests a more favorable energy alignment and reduced kinetic barrier for photoelectrochemical water oxidation, likely due to better charge collection and faster interfacial hole transfer enabled by the MXene layer. To further clarify the individual contribution of alkaline etching, a control experiment was conducted by incorporating MXene into pristine BVO without prior etching. The resulting MXene/BVO electrode exhibits a photocurrent density of 5.46 mA/cm^2^ at 1.23 V vs RHE, as shown in Figure S3 in the SI. Although this value is higher than that of pristine BVO (4.65 mA/cm^2^), it remains lower than that of the MXene/E-BVO photoanode (6.15 mA/cm^2^), indicating that alkaline etching contributes positively to PEC performance beyond what is achieved by MXene alone. This enhancement is attributed to the increased surface defect density and improved catalytic site availability introduced during etching, which facilitate hole accumulation and interfacial reaction kinetics. Meanwhile, MXene provides a conductive pathway for efficient hole extraction. The higher performance of MXene/E-BVO compared to both E-BVO and MXene/BVO confirms the synergistic effect of combining surface restructuring with interfacial engineering, resulting in more efficient charge separation, reduced recombination, and improved water oxidation efficiency. Applied bias photon-to-current efficiency (ABPE) plots were derived from the LSV curves using eq 2 and are presented in Figure 5c.^52^

2 $$\mathrm{ABPE}=\frac{J\times \left(1.23-V_{\mathrm{bias}}\right)}{P_{\mathrm{in}}}\times 100\%$$

The highest ABPE values for BVO, E-BVO, MXene/BVO and MXene/E-BVO were found to be 1.67% at 0.72 V_RHE_, 1.79% at 0.71 V_RHE_, 2.16% at 0.74 V_RHE_ and 2.52% at 0.75 V_RHE_, respectively. The MXene/E-BVO photoanode clearly achieves the best PEC efficiency, which can be attributed to the synergistic effect of alkaline etching and MXene incorporation. While etching generates more surface defect sites that serve as hole traps and reaction centers, the presence of MXene enhances conductivity and facilitates efficient hole transport, jointly contributing to improved solar-to-chemical energy conversion efficiency.

**Table 1:** Electrochemical Parameters of BVO, E-BVO, MXene/BVO and MXene/E-BVO^a^

**electrode**	**photocurrent density** (mA/cm^**2**^ **@1.23 V**_**RHE**_ **)**	**onset potential (V**_**RHE**_ **)**	***R***_**CT**_ **(Ω)**	**carrier density (cm** ^–**3**^ **)**
BVO	4.65 ± 0.18	0.25	244.6	1.72 × 10^23^
E-BVO	5.13 ± 0.21	0.31	192.6	1.68 × 10^24^
MXene/BVO	5.88 ± 0.14	0.22	180.2	2.58 × 10^24^
MXene/E-BVO	6.15 ± 0.32	0.20	116.6	4.12 × 10^24^

a Three electrodes are tested to ensure reproducibility.

Electrochemical impedance spectroscopy (EIS) was carried out at 1.23 V_RHE_ under illumination, and the Nyquist plots are displayed in Figure 5d. The fitting was conducted using the equivalent circuit model shown in the inset.^31^ Table 1 shows the charge-transfer resistance (*R*_CT_) values of BVO, E-BVO, MXene/BVO and MXene/E-BVO. The *R*_CT_ values extracted from fitting are 244.6 Ω for BVO, 192.6 Ω for E-BVO, 180.2 Ω for MXene/BVO and 116.6 Ω for MXene/E-BVO. The reduction in *R*_CT_ from BVO to E-BVO reflects improved interfacial conductivity after etching, while the lowest *R*_CT_ in MXene/E-BVO highlights the contribution of MXene to accelerating charge transfer at the electrode/electrolyte interface. This reduction in resistance plays a crucial role in minimizing energy loss during PEC operation and contributes directly to higher photocurrent and ABPE values. To further analyze bulk charge properties, Mott–Schottky analysis was performed and the plots are shown in Figure 5e. The carrier densities were calculated from the slope of the linear regions using the Mott–Schottky equation.^53^ Table 1 shows the carrier densities of BVO, E-BVO, MXene/BVO and MXene/E-BVO. The extracted donor concentrations are 1.73 × 10^23^, 1.68 × 10^24^, 2.58 × 10^24^ and 4.12 × 10^24^ cm^–3^ for BVO, E-BVO, MXene/BVO and MXene/E-BVO, respectively. The nearly 10-fold increase in carrier density for E-BVO confirms that alkaline treatment introduces donor-like surface states, while the additional increase upon MXene incorporation demonstrates that MXene also contributes to enhancing charge density, likely by improving electron mobility and maintaining higher levels of surface charge accumulation. The enhanced photoelectrochemical performance of the MXene/E-BVO electrode can be further correlated with the structural changes observed by XRD. A subtle shift in the (121) diffraction peak is detected for the MXene/E-BVO sample, suggesting the presence of interfacial lattice strain induced by interactions between MXene nanosheets and the BVO lattice. This strain may alter the local electronic environment, modify band bending at the interface, and facilitate charge separation and transport. These effects are supported by electrochemical data, where MXene/E-BVO shows the lowest charge-transfer resistance (116.6 Ω) and the highest donor density (4.12 × 10^24^ cm^–3^), as well as the highest photocurrent density (6.15 mA/cm^2^ at 1.23 V vs RHE). Such improvements indicate more efficient charge extraction and suppressed recombination, which are consistent with lattice strain–induced interfacial enhancement. Therefore, the structural distortion observed via XRD is not merely a crystallographic artifact but a contributing factor to the improved PEC performance of the MXene-modified photoanode. Finally, the long-term operational stability of the photoanodes was evaluated through chronoamperometric measurements over 10,000 s at 1.23 V_RHE_ under AM 1.5G illumination (Figure 5f). Photocurrent retention values of 74.8, 80.6, 90.4, and 93.4% were recorded for BVO, E-BVO, MXene/BVO and MXene/E-BVO, respectively. The higher stability of MXene/E-BVO highlights the dual role of MXene not only in enhancing PEC activity but also in mitigating photocorrosion and suppressing performance degradation over time. The combination of morphological tuning via alkaline etching and interfacial engineering via MXene incorporation provides a viable strategy to achieve stable and efficient PEC water oxidation. Postcatalysis characterizations for MXene/E-BVO, including SEM and XRD, are shown in Figure S4 in the SI. The morphology and crystallinity of MXene/E-BVO remain largely unchanged before and after the stability test. These results demonstrate the excellent stability of MXene/E-BVO as a photocatalyst for the oxygen evolution reaction

The enhanced photoelectrochemical performance of the MXene/E-BVO system can be primarily attributed to the improved charge-transfer dynamics enabled by the MXene layer. Upon illumination, BVO absorbs visible light and generates electron–hole pairs. The photogenerated electrons are transported through the E-BVO matrix toward the FTO substrate, while the holes migrate to the surface. In the absence of surface engineering, holes at BVO surface are prone to recombination due to limited mobility and trap states. Alkaline etching introduces surface defects that act as hole traps and catalytically active sites, facilitating interfacial water oxidation. Importantly, the incorporation of MXene forms an ultrathin, conductive network on BVO surface that significantly accelerates hole extraction. Due to its high electrical conductivity and the presence of functional surface groups, MXene provides a favorable energy alignment for hole transfer and also acts as an electron-blocking layer, minimizing back recombination. This dual effect promotes spatial charge separation. Holes are rapidly shuttled from the BVO bulk to the surface and then into water oxidation reactions, while electrons move toward the FTO substrate. Furthermore, the close interfacial contact between MXene and E-BVO may introduce local electric fields or strain-induced band bending, further assisting carrier separation.

The schematic illustration of the MXene/E-BVO photoanode architecture and its associated charge transfer mechanism is shown in Scheme 2. The configuration consists of a FTO glass substrate, a photoactive E-BVO layer, and an ultrathin MXene coating deposited on the surface. Upon illumination, BVO absorbs incident photons and generates electron–hole pairs. The photogenerated electrons are transported downward through the E-BVO matrix and collected by the FTO substrate, while holes migrate toward the surface to drive the water oxidation reaction. Alkaline etching induces the formation of abundant surface defects on E-BVO, which serve as catalytically active sites and efficient hole traps, thereby improving the likelihood of interfacial redox events. Simultaneously, the conductive MXene layer provides a favorable pathway for hole transport by bridging multiple active sites and suppressing surface recombination. This dual modification, via surface activation and interfacial engineering, not only promotes directional carrier separation but also enhances charge mobility and extraction efficiency. Consequently, this engineered system exhibits significantly improved photoelectrochemical performance, attributed to the synergistic effects of enhanced catalytic activity, reduced recombination, and efficient charge collection across the MXene/E-BVO/FTO interface.

**Scheme 2:**
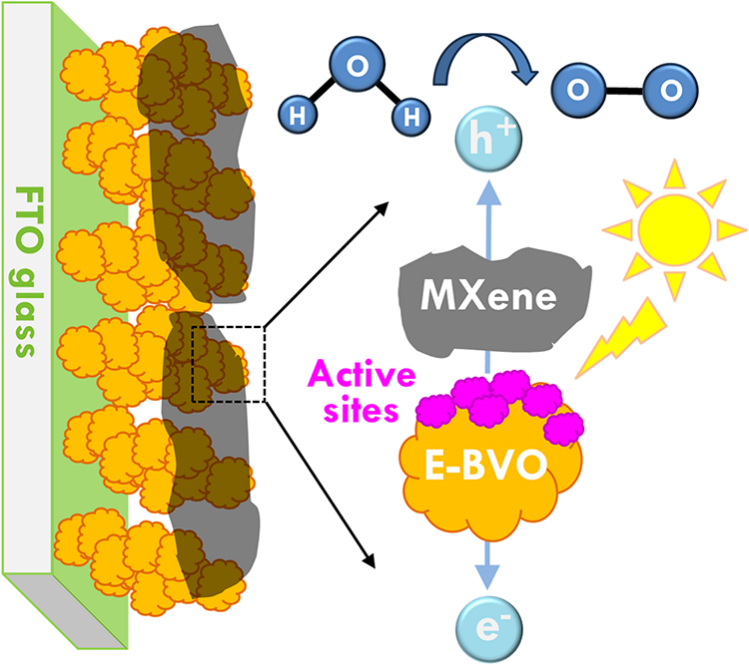
Illustration of Configuration and Charge-Transfer Paths for MXene/E-BVO System

## 4 Conclusions

In this study, a synergistic modification strategy combining alkaline etching and MXene incorporation was applied to BVO for the first time to develop an efficient PEC catalyst for water oxidation. Alkaline etching effectively introduced surface defects that serve as electroactive sites, facilitating hole accumulation and enhancing the kinetics of water oxidation. Concurrently, the introduction of MXene significantly improved the electrical conductivity of the composite, forming conductive networks that promote rapid charge transport and reduce interfacial recombination. The optimized MXene/E-BVO photoanode achieved a high photocurrent density of 6.15 mA/cm^2^, a low charge transfer resistance of 116.6 Ω, and a remarkably high carrier density of 4.12 × 10^2^ cm^–3^. Additionally, the photocurrent retention of 93.4% after 10,000 s of continuous illumination underscores its enhanced operational stability. These findings demonstrate the effectiveness of combining surface and interface engineering to overcome intrinsic limitations of BVO. Future efforts may focus on fine-tuning the MXene layer in terms of thickness, phase composition, or spatial integration, as well as exploring alternative surface engineering strategies in tandem with alkaline treatment. Such developments could further optimize charge separation and interfacial charge dynamics toward scalable, durable solar fuel generation platforms.

## Supplementary Material



## Notes

Experiments, a photo for measurement setup, TEM and LSV of MXene/BVO, SEM and XRD of MXene/E-BVO before and after stability test (PDF)
