# Supplementary material for: Boosting Photoelectrochemical Catalytic Ability of Bismuth Vanadate toward Water Oxidation via Synergistic Surface Defect Engineering and MXene-Assisted Charge Transport
Source: ACS Omega. 2025 Aug 27;10(35):40557–66. doi: 10.1021/acsomega.5c06250 (PMC12423830; doi:10.1021/acsomega.5c06250)
Supplement: Supplementary file 1 [file ao5c06250_si_001.pdf]

Supporting Information (SI)

Tsai-Mu Cheng[a,b,c*], Yi-Ru Wang[d], Yu-Hsuan Chiu[d], Chutima Kongvarhodom[e], Muhammad Saukani[f], Sibidou Yougbaré[g], Hung-Ming Chen[h], and Lu-Yin Lin[d*]

[a]Graduate Institute for Translational Medicine, College of Medical Science and Technology, Taipei Medical University, Taipei 11031, Taiwan

[b]Taipei Heart Institute, Taipei Medical University, Taipei 11031, Taiwan

[c]Cardiovascular Research Center, Taipei Medical University Hospital, Taipei 11031, Taiwan

[d]Department of Chemical Engineering and Biotechnology, National Taipei University of Technology, Taipei, Taiwan

[e]Department of Chemical Engineering, King Mongkut's University of Technology Thonburi, 126 Pracha-u-thit, Toong-kru, Bangkok 10140, Thailand

[f]Department of Mechanical Engineering, Faculty of Engineering, Universitas Islam Kalimantan MAB, Jl. Adhyaksa No. 2, Banjarmasin 70124, Indonesia

[g]Institut de Recherche en Sciences de la Santé (IRSS-DRCO)/Nanoro, 03 B.P 7192, Ouagadougou 03, Burkina Faso

[h]Gingen technology Co., LTD., Rm. 7, 10F., No.189, Sec. 2, Keelung Rd., Xinyi Dist., Taipei 11054, Taiwan

[*]Corresponding author (T.M. Cheng): E-mail: tmcheng@tmu.edu.tw

[*]Corresponding author (L.Y. Lin): E-mail: lylin@ntut.edu.tw

Synthesis of BVO film on FTO glass

Firstly, the BiOI film was synthesized using electrodeposition, serving as the precursor for BVO. The conductive substrate used was fluorine-doped tin oxide (FTO) glass. The electrodeposition electrolyte consisted of two separate solutions. One solution contained 3.32 g of KI (Showa, 99.5%) and $Bi(NO_3)_3 \cdot 5H_2O$ (Alfa Aesar, 98%) dissolved in deionized water (DIW), with the pH adjusted to 1.7 by adding HNO_3. The other solution was prepared by dissolving 0.497 g of p-benzoquinone (Alfa Aesar, 98+%) in 20 mL of absolute ethanol. Electrodeposition was performed using a three-electrode setup, with an Ag/AgCl reference electrode and a platinum counter electrode, applying a potential of -0.1 $V_{Ag/AgCl}$ for 3 minutes. Next, the BVO electrode was fabricated by drop-casting, followed by annealing. The drop-casting solution consisted of 0.2 M vanadium acetylacetonate (Acros Organics, 99%) and 0.02 M sodium tungstate (Acros Organics, 99+%) in 5 mL of dimethyl sulfoxide (DMSO, J.T.Baker, 99.9%). This solution was applied to the BiOI/FTO glass substrate, and the coated sample was annealed at 450°C for 2 hours at a heating rate of 2°C/min. After annealing, the electrode was rinsed with a 1 M NaOH solution (Fluka, 98+%) to eliminate any residual V_2O_5.

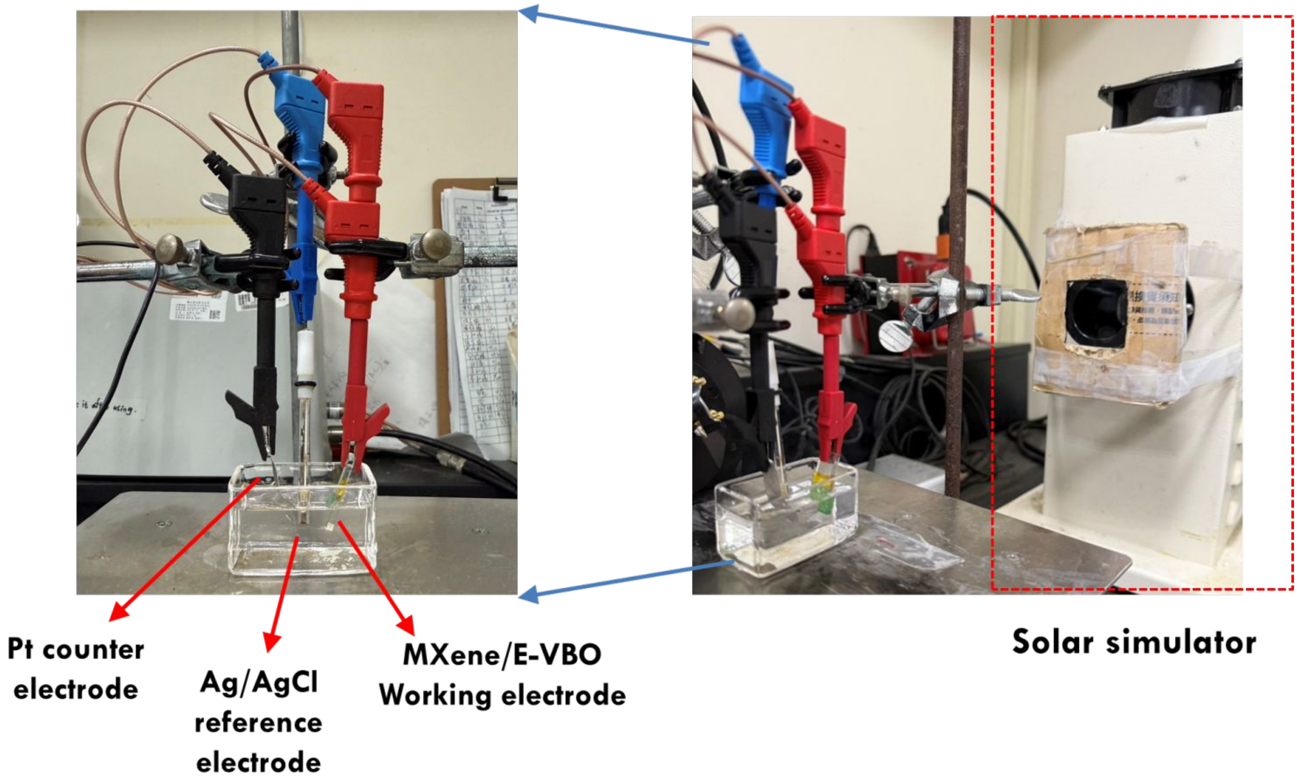

Figure S1 The photo of electrochemical measurement setup.

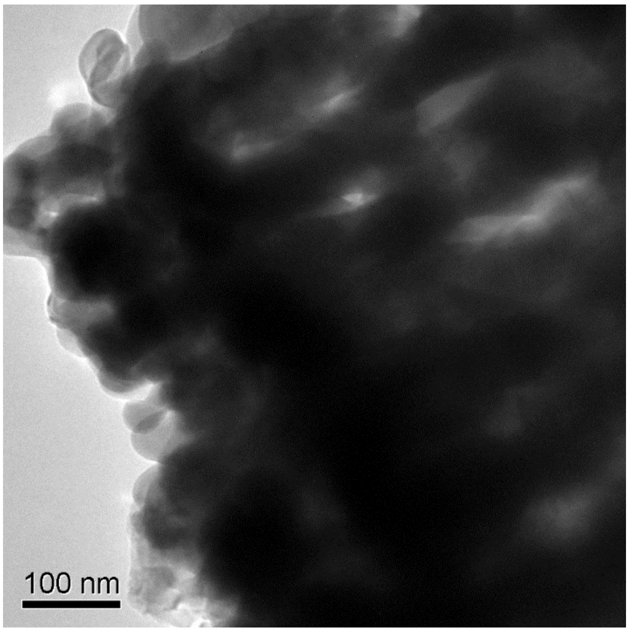

Figure S2 The TEM image of MXene/E-BVO.

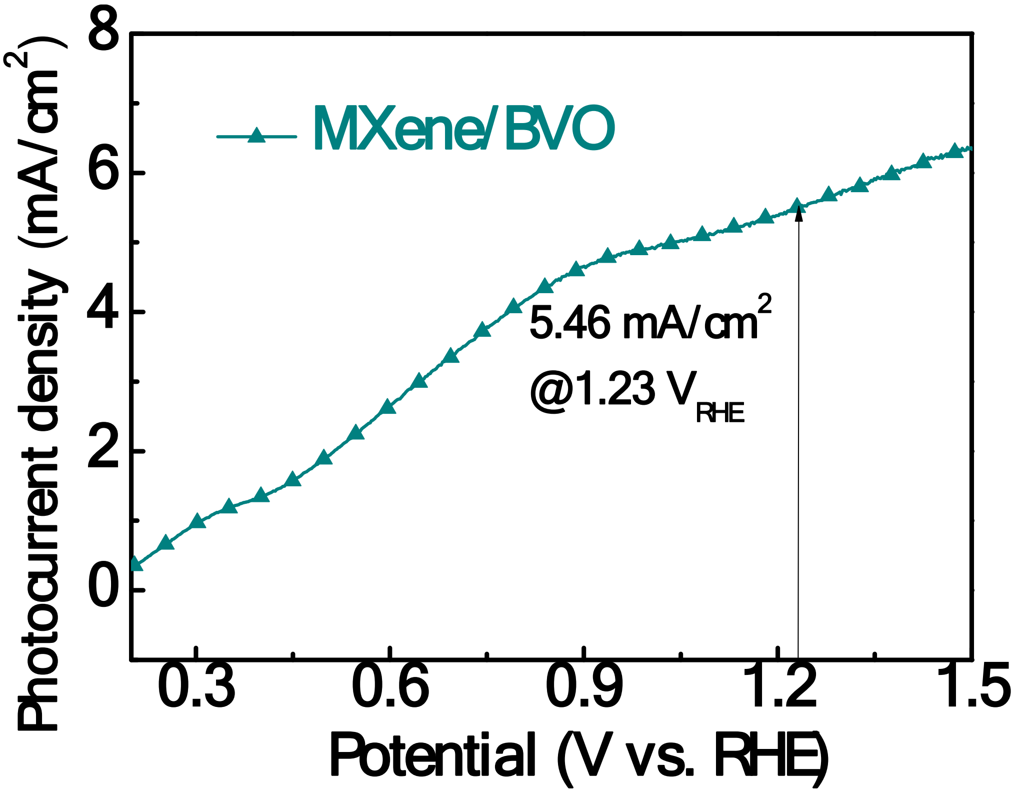

Figure S3 The LSV curve of the MXene/BVO electrode.

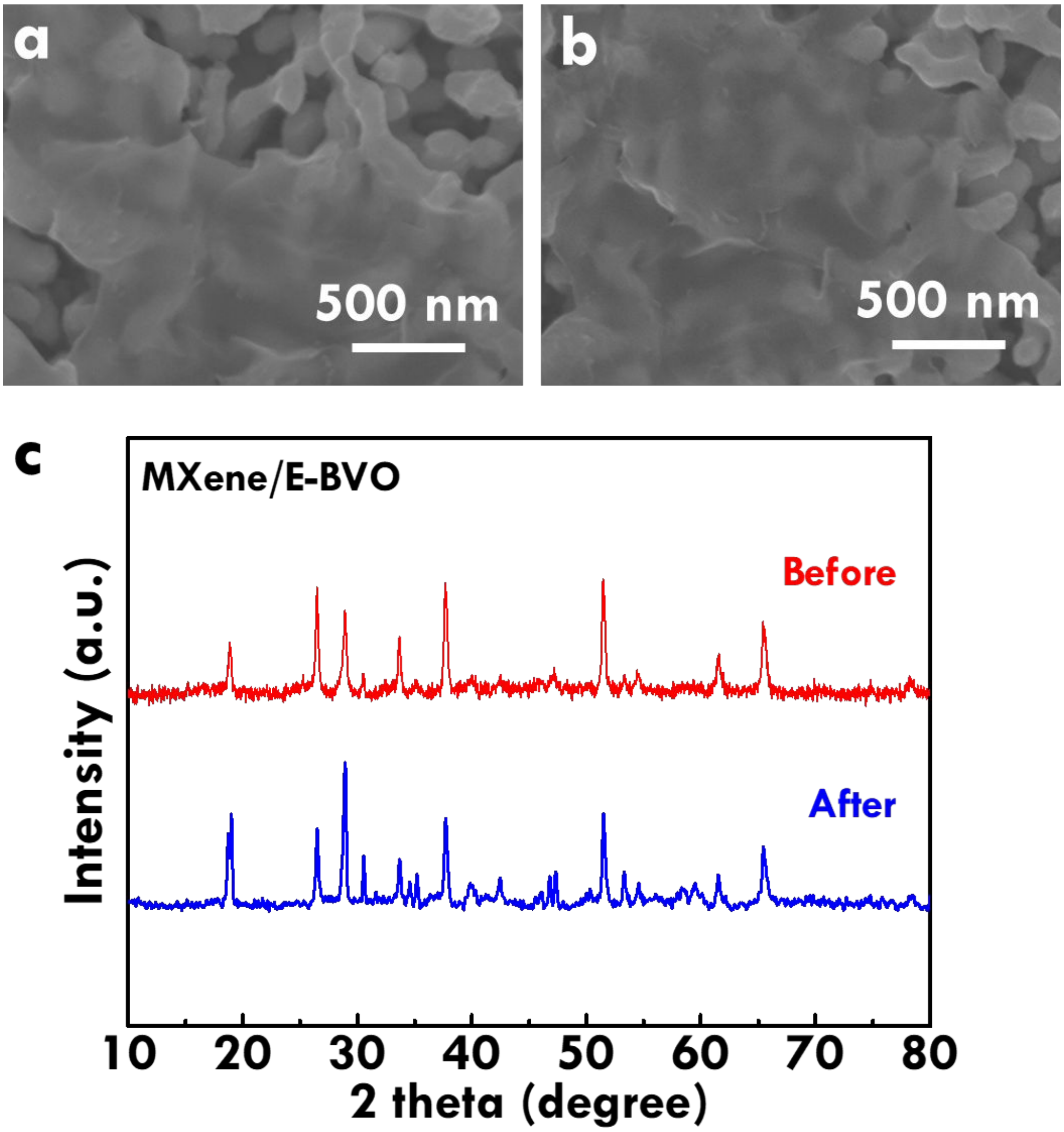

Figure S4 The SEM images (a) before and (b) after stability test; (c) XRD patterns of MXene/E-BVO before and after stability test.
